# Assessing Mental Health Conditions in Women with Gestational Diabetes Compared to Healthy Pregnant Women

**DOI:** 10.3390/healthcare12141438

**Published:** 2024-07-19

**Authors:** Keren Grinberg, Yael Yisaschar-Mekuzas

**Affiliations:** Department of Nursing Sciences, Faculty of Social and Community Sciences, Ruppin Academic Center, Emek Hefer 402500, Israel; yaeli@ruppin.ac.il

**Keywords:** gestational diabetes mellitus (GDM), anxiety, distress, depression, somatization

## Abstract

Introduction: Pregnant women with gestational diabetes mellitus (GDM) experience higher psychological stress levels than healthy pregnant women. The objectives of the current study were to examine (1) the differences in anxiety, depression, stress, and somatization levels between women diagnosed with GDM and healthy pregnant women, and (2) the differences in anxiety, depression, stress, and somatization levels among women with well-controlled blood sugar levels compared to those who are not well controlled. Methods: A quantitative cross-sectional study was conducted, involving 103 women who had been pregnant at least once, including 40 women diagnosed with GDM and 63 healthy pregnant women. An online questionnaire was distributed that included three parts: socio-demographic parameters, the DASS-21 questionnaire assessing anxiety, depression, and stress, and the Brief Symptom Inventory (BSI) questionnaire assessing somatization. Results: Differences in the anxiety (t = 14.470, <0.001), depression (t = 8.17, <0.001), stress (t = 16.354, <0.001), and somatization (t = 13.679, <0.001) levels between women diagnosed with GDM and healthy pregnant women were found. Women diagnosed with GDM reported higher levels of anxiety, depression, stress, and somatization compared to those without GDM. Additionally, women with better blood sugar control, as indicated by lower glycated hemoglobin (HbA1c) levels had lower anxiety (t (38) = −2.04, *p* < 0.05), depression (t(38) = −2.88, *p* < 0.01), stress (t(38) = −1.88, *p* < 0.05), and somatization (t(38) = −1.88, *p* < 0.05) levels compared to women with poorer blood sugar control. Conclusions: Pregnant women diagnosed with GDM report higher levels of negative mental health conditions such as anxiety, depression, stress, and somatization compared to healthy pregnant women.

## 1. Introduction

Gestational diabetes mellitus (GDM) stands out as a prevalent complication during pregnancy, affecting approximately 14% of pregnant women globally [1]. This condition manifests when women, not previously identified as having diabetes, develop chronic hyperglycemia during their pregnancy. The risks associated with GDM encompass microvascular and macrovascular complications, posing threats to the small blood vessels, nerves, as well as macrovascular issues like the heart and blood vessel diseases. The interplay of genetics has been established as a contributing factor to diabetes development and its associated risks [2,3]. Given its diagnosis during pregnancy, GDM holds the potential to adversely impact both maternal and fetal health [4].

The American Diabetes Association classifies GDM as first-time diabetes detected during the second or third trimester of pregnancy, although its classification under type 1 or type 2 diabetes is not entirely clear [5]. The risk factors for GDM include overweight or obesity, advanced maternal age, and a family history of diabetes or chronic insulin resistance. Long-term repercussions for the offspring include an elevated risk of obesity, type 2 diabetes, and cardiovascular diseases [6,7,8].

The diagnosis of GDM escalates the risk of emotional distress, encompassing depression, anxiety, or stress. Furthermore, it negatively influences the self-perception of health and quality of life among diagnosed women [9]. Research indicates a prevalence of depressive symptoms ranging from 25.9% to 56.7%, and anxiety symptoms from 4.8% to 57.7%, among women with GDM [10].

In a study by Lee et al. [9], it was found that 40% of women with GDM reported experiencing anxiety, while a tenth exhibited symptoms of depression and stress. Fu et al. [11] observed that up to 60.8% of women with GDM might suffer from anxiety, with some distress attributed to a lack of knowledge about GDM and some to concerns about fetal health [12]. A Malaysian study reported a prevalence of 12.5% for depression symptoms, 39.9% for anxiety, and 10.6% for stress among women with GDM [9]. Additionally, mental distress can manifest physically, with somatization acting as a defense mechanism linked to depression and anxiety, where individuals avoid intolerable feelings and fantasies through physical symptoms [6]. A study involving 62 pregnant women (second/third trimester) revealed a correlation between pregnancy and personal disorders like somatization, depression, and anxiety, influenced by socio-demographic characteristics and the pregnancy process itself [13].

Understanding the emotional and personality aspects and their relation, respectively, to the individual’s reaction to GDM and the GDM diagnosis is crucial for tailoring effective treatment for each pregnant women diagnosed with GDM.

While existing studies have linked personality traits and mental health conditions to clinical measures in diabetic patients, mental health conditions such as anxiety, depression, stress, and somatization have not been adequately explored among pregnant women diagnosed with GDM in comparison to a control group of healthy pregnant women. Hence, the current study’s aims are to (1) investigate the differences in the anxiety, depression, stress, and somatization levels between pregnant women with and without GDM; and (2) assess the variations in the levels of anxiety, depression, stress, and somatization among women with well-controlled diabetes versus those with poorly controlled diabetes.

### Research Hypotheses

**H1.**  *Significant differences will be identified in the levels of anxiety, depression, stress, and somatization between women diagnosed with GDM and healthy pregnant women. It is anticipated that higher levels of anxiety, depression, stress, and somatization will be observed among women with GDM.*

**H2.**  *Significant differences will be observed in the levels of anxiety, depression, stress, and somatization among women with well-controlled diabetes, based on the HbA1c levels, in comparison to women with poorly controlled diabetes. It is expected that higher levels of anxiety, depression, stress, and somatization will be evident among women with uncontrolled blood sugar levels.*

## 2. Methods

This was a quantitative cross-sectional study involving a sample of 103 women who had given birth at least once, comprising 40 women diagnosed with GDM and 63 healthy pregnant women. The research utilized an online questionnaire encompassing three sections: (1) a socio-demographic questionnaire, (2) the DASS-21 questionnaire [14] evaluating anxiety, depression, and stress, and (3) the Brief Symptom Inventory (BSI) assessing somatization [15].

### 2.1. Tools

Socio-demographic section: This segment assessed participants’ demographic background through inquiries about age, education, ethnicity, number of children at home, births and pregnancies, youngest child’s age, economic status, smoking and alcohol habits, physical activity, healthy food habits and family history of diabetes.

The DASS-21 (Depression, Anxiety, and Stress Scale) questionnaire [14] was translated into Hebrew by Janine Lurie. This self-report questionnaire gauges the frequency of symptoms in three domains: depression, anxiety, and stress. It consists of 21 items across three scales: depression, anxiety, and stress, each containing 7 items, which were rated by the participants. The questionnaire has shown good psychometric properties, including good internal consistency in both clinical and general populations (Cronbach’s alpha: 0.88 for the Depression Scale, 0.82 for the Anxiety Scale, 0.9 for the Stress Scale, and 0.93 for the total score). The questionnaire has also demonstrated good validity and its scores have shown high correlations with other measures of depression and anxiety levels [14,16,17].

The questionnaire is scored by summing the participant’s ratings for each scale, based on the item distribution across the different scales. 

Anxiety Scale: The items assess various expressions of anxiety, including somatic anxiety (physical symptoms), situational anxiety (anxiety related to specific situations), and subjective experience of anxiety (personal perception of anxiety). The scale ranges from 0 (never) to 3 (almost always) for each item (items: 2, 4, 7, 9, 15, 19, 20). Higher scores indicate increased levels of anxiety, providing a quantitative measure of anxiety severity across these different dimensions.

Distress Scale: Items measuring various expressions of distress and pressure, both chronic and situational, are found to be related to generalized anxiety symptoms, including emotional arousal, difficulty calming down, and a tendency to react with anger, irritability, impatience, and intolerance. The scale range is between 0 (never) and 3 (almost always) (items: 1, 6, 8, 11, 12, 14, 18), and higher scores indicate a higher level of perceived distress (average of all the other dependent variables).

Depression Scale: Items assess different expressions of depression, including dysphoria, self-image disturbance, lack of hope, anhedonia, and inertia. The scale range is between 0 (never) and 3 (almost always) (items: 3, 5, 10, 13, 16, 17, 21), and higher scores indicate elevated depression levels.

Brief Symptom Inventory (BSI) somatization questionnaire: The items (13) describe various complaints or symptoms, some of which are related to pain, such as chest pain, headaches, lower back pain, and others to unpleasant sensations in the body, such as pressure, numbness, swelling, nausea, and dizziness. The scale frequency of symptom experience in the past month [15] is between 0 (not at all) and 4 (very much). A high score on this questionnaire indicates a high frequency of somatic symptoms. The questionnaire has been translated into Hebrew and has good reliability (α = 0.850) and high internal consistency and validity [18].

### 2.2. Research Procedure

This study was approved by the Ethics Committee of the Faculty of Social and Community Sciences at the Ruppin Academic Center. Using a snowball sampling method, an online questionnaire was distributed through social networks such as Facebook, targeting various women’s groups and forums for mothers and pregnant women. Participants included women diagnosed with GDM and healthy pregnant women, aged between 18 and 50 years.

### 2.3. Data Analysis

IBM SPSS Statistics 28 software was used for the statistical analysis. Descriptive statistics were used to outline the demographic and research variables. For testing the first research hypothesis, an independent *t*-test was conducted for anxiety, depression, stress, and somatization. The second hypothesis involved a dichotomous division based on balanced and unbalanced HbA1C levels, with an independent *t*-test conducted for anxiety, depression, stress, and somatization.

## 3. Results

**Figure 1:**
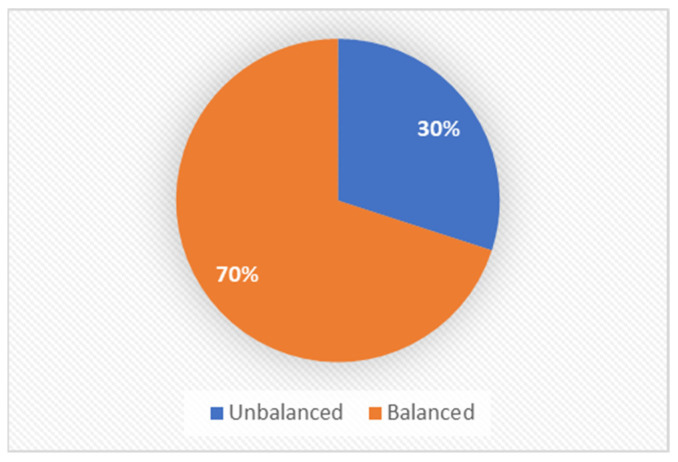
Balanced and unbalanced women with GDM.

In this quantitative study, a sample of 103 pregnant women (at least once) was analyzed, including 40 diagnosed with GDM and 63 healthy pregnant women. The participants’ average age was 34.24 years. The marital status distribution indicated that 79% were married, 14% were divorced, 3% were single, and 4% defined their status as “other”. The youngest child’s age ranged from six months to 13 years, with an average age of 3.55. The family size varied, with 38% having one child, 23% having two children, 28% having three children, 7% having four children, 2% having five children, and an additional 2% having six children. The educational backgrounds included 11% with a high school education, 33% beyond high school, 42% with a bachelor’s degree, 13% with a master’s degree, and 12% holding a doctoral degree. The religious affiliation comprised 77% Jewish, 11% Christian, 9% Muslim, and others identifying as non-religious. Among the diabetic patients, 70% considered themselves balanced, and 30% unbalanced (Figure 1). Regarding lifestyle, 73% never smoked, 13% smoked occasionally, 9% smoked frequently, 1% smoked very frequently, and 4% smoked regularly. The physical activity engagement showed that 8% never engaged, 32% occasionally, 19% frequently, 24% very frequently, and 17% always engaged. The latest glycated hemoglobin (HbA1C) values in the blood test ranged from 6.3 to 7.23, with an average of 6.73.

**Figure 2:**
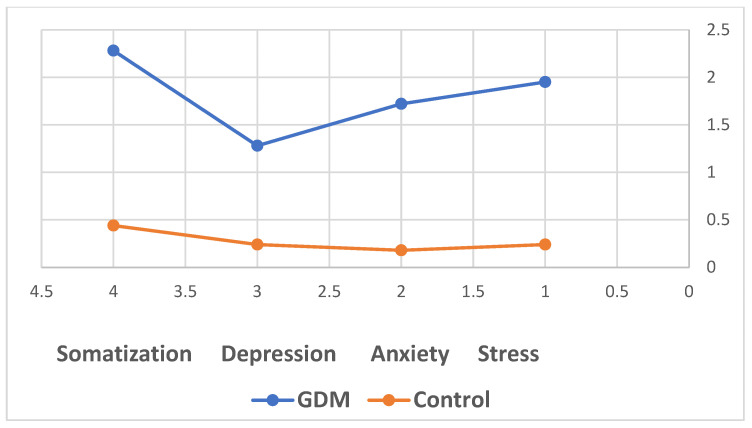
Differences in the levels of somatization, depression, anxiety and stress among pregnant women with GDM vs. control.

The first research hypothesis was confirmed, revealing significant differences in the levels of anxiety (t = 14.470, *p* < 0.001), depression (t = 8.17, *p* < 0.001), stress (t = 16.354, *p* < 0.001), and somatization (t = 13.679, *p* < 0.001) between women diagnosed with GDM and healthy pregnant control group (Figure 2).

To explore the differences in anxiety, depression, stress, and somatization concerning diabetes control, an independent samples *t*-test demonstrated significant differences in the anxiety (t(38) = −2.04, *p* < 0.05), depression (t(38) = −2.88, *p* < 0.01), stress (t(38) = −1.88, *p* < 0.05), and somatization (t(38) = −1.88, *p* < 0.05) levels between women with well-controlled and poorly controlled diabetes. Higher levels of anxiety, depression, stress, and somatization were observed in women with poorly controlled diabetes.

**Figure 3:**
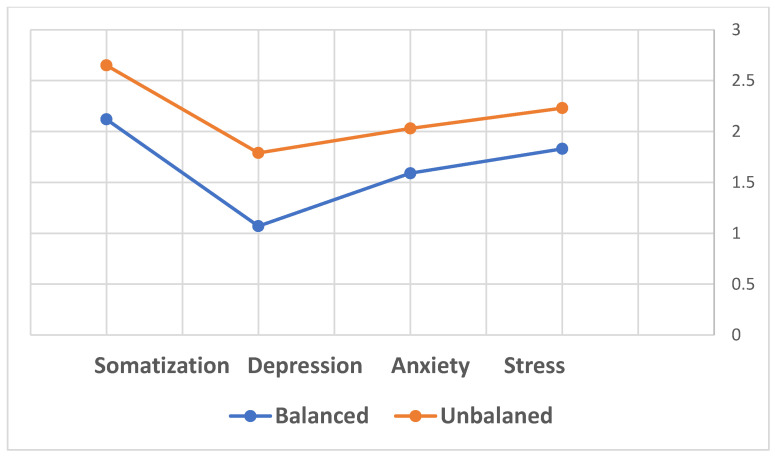
Differences in the levels of somatization, depression, anxiety and stress among balanced women with GDM vs unbalanced women with GDM.

The second research hypothesis was confirmed, indicating that women with better HbA1C levels exhibited lower levels of anxiety, depression, stress, and somatization compared to those with poorer control. Women diagnosed with GDM were asked to categorize themselves as balanced or unbalanced based on their reported HbA1C levels during pregnancy (Table 1, Figure 3). The results concerning the second hypothesis indicated that balanced women exhibited significantly lower levels of anxiety, depression, stress, and somatization compared to those who were unbalanced (Table 1, Figure 3).

In summary, the second hypothesis was confirmed, revealing lower levels of anxiety, depression, stress, and somatization in balanced women compared to those who were not balanced.

## 4. Discussion

This study delved into variations in the levels of anxiety, depression, stress, and somatization among women with GDM compared to healthy pregnant women, and it further explored differences based on diabetes control. Both research hypotheses were substantiated, shedding light on critical aspects of psychological well-being during pregnancy, such as the levels of anxiety, depression, stress, and somatization among women diagnosed with GDM compared to healthy pregnant women.

Aligning with the findings of OuYang et al. [10], this study established that women with GDM exhibit heightened levels of anxiety and depression compared to healthy pregnancies.

Expanding on this comprehension, we aim to investigate the notable disparities and heightened levels of somatization among women with GDM in comparison to those without it, a facet that, to the best of our knowledge, has not previously been examined.

The psychological pressure experienced by women with GDM, driven by the awareness of the potential complications for both the pregnancy and the fetus due to uncontrolled diabetes, might contribute to possibly elevated antenatal depression, anxiety, and stress. 

Furthermore, it was found that 40% of women who had GDM suffered from anxiety, and a tenth of them suffered from symptoms of depression and stress, and these findings align with earlier research [9], emphasizing the importance of addressing psychological well-being in this demographic.

Collier et al.’s insights regarding economic difficulties, healthcare and food costs, as well as physical barriers, add a socio-economic dimension to the challenges faced by women with GDM [19]. These women also experienced difficulties in finding available, valid, and relevant information, as well as in communication with the healthcare system to obtain such information. The perceived time-consuming nature of insulin injections reflects additional psychological burdens. These findings underscore the multifaceted nature of the challenges this population encounters, necessitating holistic intervention strategies [20].

Accumulating evidence underlines the increasing potential adverse effects of emotional distress during pregnancy on infant development, including depression, anxiety, general stress, and specific pregnancy-related stress [21,22,23]. Pregnant women diagnosed with GDM experience heightened psychological pressure due to the awareness of uncontrolled diabetes, which can lead to pregnancy-related complications and harm the fetus. This emphasizes the tendency to develop prenatal depression, anxiety, and stress [11].

The second part of this study reaffirmed that a perceived balance in diabetic pregnant women correlates with lower levels of anxiety, depression, stress, and somatization. This suggests that a sense of control over the disease reduces negative emotions, such as feelings of stress and anxiety, and their negative consequences. The treatment strategies for GDM, including blood glucose management and specialized obstetric care, appear effective in lowering the risk of perinatal complications. The interaction of psychosocial well-being with diet and physical activity highlights the interconnectedness of lifestyle factors and glycemic control. Thus, awareness can very likely reduce health risks, including the fetus-related risks, and lead to decreased levels of anxiety, stress, depression, and somatization. 

This study underscores the need for healthcare providers to assess psychological well-being and distress at the time of GDM diagnosis and to help control their glycemic balance through a balanced diet and regular physical activity [24]. It is also possible that the woman’s perception of the disease is related to her levels of anxiety, stress, depression, and somatization. For example, mental health is an important factor in the management of GDM that is still underestimated and should be implemented into daily practice [25]. Hence, in order to optimize the treatment strategies for GDM patients and alleviate the burden of the disease, care providers need to assess the psychological distress of their patients at the time of GDM diagnosis.

The influence of coping resources, both personal and professional, on the levels of stress, anxiety, depression, and somatization merits further exploration. Unfortunately, the current study did not assess the woman’s knowledge about her condition or her perception of the illness and sense of well-being. However, evaluating women’s knowledge about their well-being and condition and their perception of illness is crucial for a comprehensive understanding of the emotional experiences associated with GDM and can most likely have a direct relation. In addition, a study by Hjelm et al. [26] found that beliefs differed and were related to the healthcare model chosen. As found, women with GDM (monitored at a specialist maternity clinic) believed that it is only a transient condition during pregnancy, and they expressed fear about the future risk of developing type 2 diabetes.

In summary, the current study provides valuable insights into the mental health conditions of pregnant women with and without GDM. The findings underscore the importance of addressing psychological well-being, especially in the context of GDM, and indicate that pregnant women diagnosed with GDM report higher levels of negative mental health conditions such as anxiety, depression, stress, and somatization compared to healthy pregnant women. Furthermore, women diagnosed with GDM with balanced levels of HBA1C reported lower levels of anxiety, depression, stress, and somatization compared to women with non-balanced sugar levels. Recommendations include holistic interventions, further research on a larger and diverse population, and exploration of the factors influencing emotional states in women with GDM.

## 5. Limitations

This study included a relatively small sample of pregnant women, both with and without GDM. This limited sample size raises concerns about the generalizability of the results to the broader population of pregnant women. The findings might not fully encompass the varied experiences within this demographic, so it is advisable to exercise caution when extrapolating the outcomes.

The study’s reliance on self-report questionnaires introduces the potential for participant bias. Responses to questions related to anxiety, depression, stress, and somatization may be influenced by subjective interpretations and individual perspectives. Participants might provide socially desirable responses or may not accurately represent their psychological states, impacting the reliability of the data. In addition, the unequal representation of women with GDM and healthy pregnant women raises concerns about the representativeness of the study cohort. A more balanced sampling approach would have strengthened the study’s external validity by ensuring a more equitable comparison group.

This study did not delve into certain crucial factors that could influence emotional states, such as participants’ knowledge about their condition, perception of illness, coping mechanisms, and sense of well-being. These unexplored variables could significantly contribute to the understanding of the emotional experiences of pregnant women with GDM. In addition, this study primarily focused on anxiety, depression, stress, and somatization, leaving other potential psychological aspects unexplored. Comprehensive psychological assessments encompassing a broader range of variables would offer a more nuanced understanding of the emotional well-being of pregnant women with GDM. The cross-sectional design employed in this study captures a snapshot of participants’ experiences at a specific point in time. Longitudinal studies would provide a more dynamic view, allowing for the examination of changes in emotional states over the course of pregnancy, potentially uncovering patterns or fluctuations.

## 6. Conclusions

The findings of this study shed light on the psychological challenges faced by pregnant women diagnosed with GDM. The following conclusions can be drawn from the study.

### 6.1. Heightened Psychological Concerns in GDM Patients

This study underscores the increased prevalence of psychological issues among pregnant women with GDM. The higher levels of anxiety, depression, stress, and somatization observed in this group emphasize the need for targeted interventions to address their unique mental health needs.

### 6.2. Call for Active Psychological Interventions

This study highlights the importance of implementing active and effective psychological intervention measures for pregnant women with GDM. Given the psychological pressures associated with GDM, tailored interventions could contribute to better mental health outcomes during pregnancy. Healthcare providers should consider incorporating psychological support into routine care for this population.

### 6.3. Focus on Pregnancy Safety and Outcomes

Recognizing the psychological impact on pregnancy outcomes, this study suggests that addressing the mental health of GDM patients is crucial not only for their well-being but also for achieving better pregnancy outcomes. Interventions aimed at reducing anxiety, depression, stress, and somatization may contribute to safer and healthier pregnancies for women with GDM.

### 6.4. Need for Further Research

While this study contributes valuable insights into the psychological aspects of GDM, the identified limitations underscore the need for cautious interpretation. Future research endeavors should address these constraints, employing larger and more diverse samples, incorporating objective measures, and exploring a broader array of contributing factors to enhance the robustness of the findings. 

Future research should focus on identifying and examining the various factors that influence the levels of anxiety, stress, depression, and somatization among women diagnosed with GDM. Factors such as socio-economic status, cultural considerations, medication treatment and access to healthcare should be explored to provide a more comprehensive understanding of the multifaceted nature of psychological well-being in this population.

In conclusion, addressing the psychological well-being of pregnant women with GDM is a crucial aspect of comprehensive prenatal care. This study emphasizes the imperative for proactive measures to support these women emotionally, ultimately contributing to safer pregnancies and improved outcomes. Ongoing research in this field will further refine our understanding and inform evidence-based interventions for this specific group of pregnant women.

## Figures and Tables

**Table 1:** Differences in the levels of somatization, depression, anxiety and stress between pregnant women.

Variable	T-Value	Sig
Stress	−1.878	256 (*p* < 0.05)
Anxiety	−2.041	153 (*p* < 0.05)
Depression	−2.875	542 (*p* < 0.01)
Somatization	−1.884	652 (*p* < 0.05)

## Data Availability

The original contributions presented in this study are included in the article; further inquiries can be directed to the corresponding author.
